# The cognitive cerebellum: a hub for motor, emotional, and executive control

**DOI:** 10.1055/s-0045-1814378

**Published:** 2026-01-25

**Authors:** Renata Barreto Tenório, Andressa Aline Vieira, Gustavo L. Franklin, Marcus Vinícius Della Coletta, Hélio Afonso Ghizoni Teive, Carlos Henrique Ferreira Camargo

**Affiliations:** 1 Universidade Federal do Paraná, Programa de Pós-Graduação em Medicina Interna, Disciplina de Doenças Neurodegenerativas, Curitiba PR, Brazil.; 2 Instituto de Neurologia de Curitiba, Setor de Neurogenética, Curitiba PR, Brazil.; 3 Universidade Federal do Paraná, Setor de Ciências da Saúde, Departamento de Clínica Médica, Serviço de Clínica Médica, Curitiba PR, Brazil.; 4 Pontifícia Universidade Católica do Paraná, Faculdade de Medicina, Departamento de Clínica Médica, Curitiba PR, Brazil.; 5 Universidade do Estado do Amazonas, Manaus AM, Brazil.; 6 Universidade Federal do Paraná, Setor de Ciências da Saúde, Departamento de Clínica Médica, Serviço de Neurologia, Curitiba PR, Brazil.

**Keywords:** Spinocerebellar Ataxias, Dementia, Huntington Disease, Alzheimer Disease, Cerebellum, Cerebellar Cognitive Affective Syndrome, Cognitive Dysfunction

## Abstract

For many decades, the cerebellum was regarded almost exclusively as a structure devoted to motor regulation, responsible for balance, coordination, and the fine-tuning of movement. This view began to change in the 1990s, when studies with patients with isolated cerebellar lesions revealed cognitive and affective disturbances that could not be solely explained by cortical dysfunction. Subsequent anatomical, neuroimaging, and clinical investigations demonstrated robust reciprocal connectivity between the cerebellum and prefrontal regions, while functional imaging confirmed cerebellar activation during cognitive tasks without any motor component. Cognitive impairment linked to cerebellar dysfunction is now recognized as a prominent feature of spinocerebellar ataxias, and it has also been reported in other major neurodegenerative disorders, including Huntington's disease, Parkinson's disease, and Alzheimer's disease. Therefore, the aim of the current narrative review is to synthesize and critically analyze the pathophysiological, neuropathological, genetic, clinical, and neuroimaging evidence that underscores the cerebellum's essential contributions to cognition.

## INTRODUCTION

For much of the nineteenth and twentieth centuries, the cerebellum was regarded almost exclusively as a motor structure, responsible for balance, coordination, and the fine-tuning of movement. 1 This traditional view began to shift in the late twentieth century, when clinical studies with patients 2 3 4 with isolated cerebellar lesions revealed cognitive and affective changes that could not be solely explained by cortical dysfunction. A central conceptual turning point came from the pioneering work of Henrietta Leiner, Alan Leiner, and Robert Dow. 5 6 Beginning in the 1980s, Henrietta Leiner challenged the long-standing motor-centric perspective, proposing that the dramatic evolutionary enlargement of the human cerebellum, particularly its projections through the dentate nucleus to prefrontal association cortices, endowed it with a decisive role in cognition and language. Together with Alan Leiner, she argued that the cerebellum enhances the speed and fluency of mental and linguistic processes, famously describing it as “the treasure at the bottom of the brain.” 5 6 7 Supporting these ideas, anatomical studies have demonstrated reciprocal connectivity between the cerebellum and prefrontal regions, while functional studies have confirmed cerebellar activation during purely-cognitive tasks. 1 2 3 4 5 6 7 8 9

Building on this foundation, Schmahmann and Sherman 3 4 described cerebellar cognitive affective syndrome (CCAS), a distinct constellation of deficits including executive dysfunction, language disturbances, visuospatial impairment, and personality changes. This clinical construct provided strong evidence that the cerebellum participates in a broad spectrum of higher-order processes and marked a paradigm shift in cerebellar research. 2 3 4

More recent evidence has extended this framework through convergent findings from anatomical, clinical, and advanced neuroimaging studies. Cognitive impairment is now recognized as a frequent, though heterogeneous, feature of spinocerebellar ataxias (SCAs), with considerable variability in presentation, severity, and affected domains. 10 11 Comparable associations have been reported in other neurodegenerative diseases. In Huntington's disease (HD), for example, cerebellar circuits have been implicated in cognitive dysfunction, 12 while in Parkinson's disease (PD), structural and functional imaging studies reveal that maladaptive cerebellocortical connectivity contributes significantly to non-motor symptoms, including cognitive decline. In Alzheimer's disease (AD), the cerebellum is relatively spared in the early stages, but it appears to become involved as dementia progresses. 13

This body of work establishes the cerebellum as a crucial contributor to higher-order processes—including working memory, executive function (EF), language, affect regulation, and social cognition. 2 3 4 5 6 7 8 9 13 Accordingly, the goal of the present narrative review is to synthesize and critically analyze pathophysiological, neuropathological, genetic, clinical, and neuroimaging evidence that underscores the cerebellum's essential contributions to cognition.

## METHODS

The current narrative review encompassed original research articles, including observational, cohort, cross-sectional, and case-control studies, as well as case series, clinical cases, meta-analyses, and reviews that contribute to connect genetics, clinical findings, neurophysiology, neuroimaging, and the pathophysiology of cognition with the cerebellum.

A thorough three-step search strategy was employed to retrieve relevant literature:

- Initial search: A preliminary exploration was conducted in key databases such as PubMed, Embase, and the Cumulative Index to Nursing and Allied Health Literature (CINAHL), using terms such as *cognition* , *ataxia* , and *cerebellum* . This step involved the identification of index terms, Medical Subject Headings (MeSH), and keywords from the titles and abstracts of the relevant papers retrieved. No restrictions were applied regarding the publication year or language;
- Full search: This subsequent, more extensive search incorporated all the identified keywords and index terms across the aforementioned databases;
- Hand search: The reference lists of the retrieved studies were manually reviewed to identify additional studies not captured in the database searches.

The reviewers independently screened all studies, with disagreements resolved by consensus.

## PATHOPHYSIOLOGY

**Figure 1:**
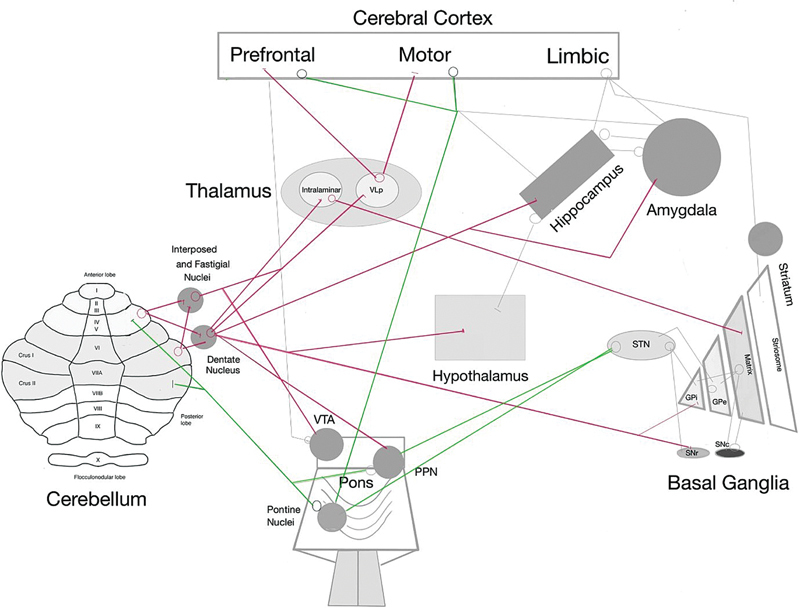
Abbreviations: GPi, globus pallidus internus; GPe, globus pallidus externus; PPN, pedunculopontine nucleus; SNc, substantia nigra pars compacta; SNr, substantia nigra pars reticulata; STN, subthalamic nucleus; VLp, ventral lateral posterior nucleus; VTA, ventral tegmental area. Note: Adapted from Xie et al., 13 Camargo et al., 99 and Kaji. 100 Simplified diagrams illustrating the main interrelationships among the cerebellum and brain structures associated with cognition. The central afferent cerebellar connections are shown in green, and the efferent connections, in red.

The mechanisms used by the cerebellum to regulate sensorimotor and vestibular functions are also applied to cognitive, emotional, and autonomic processes. This hypothesis is supported by the theories of dysmetria of thought and universal cerebellar transform, which propose that the cerebellum maintains behavior around a homeostatic, automatic, and unconscious baseline guided by implicit learning and contextual cues. 1 3 4 5 6 14 The cerebellum's functional topography enables the modulation of networks involved in various functions. 15 The sensorimotor cerebellum is represented in the anterior lobe and a second time in lobule VIII; lesions to these areas result in the cerebellar motor syndrome of ataxia, dysmetria, dysarthria, and impaired oculomotor control. The cognitive/limbic cerebellum is located in the posterior lobe, where current evidence indicates distinct topographic representations ( Figure 1 ). 15

Growing evidence of the cerebellum's role in human cognition has emerged through anatomical, clinical, and neuroimaging studies. Stoodley 9 explored the relationship between the cerebellum and cognition through neuroimaging data, showing cerebellar activation during cognitive tasks involving language, visuospatial processing, EFs, and working memory. She emphasized that the cerebellum is activated even during tasks that do not involve movement, challenging traditional views of its role limited to motor control. Specific cerebellar areas—particularly lobules VI and VII, including crus I and II—are activated in response to varying cognitive demands. This connectivity-based evidence supports a modular organization of the cerebellum related to various cognitive functions. 9 Patients with cerebellar damage confined to the posterior regions exhibited primarily cognitive dysfunctions, such as acute psychomotor retardation, reduced speech output, and mild cognitive decline, while motor functions were largely preserved. This pattern supports the model proposing that the cerebellum enhances cognitive performance through internal processing mechanisms that modulate motor and cognitive functions. 9 13

Well-established evidence indicate that the sensorimotor regions of the cerebellum maintain extensive reciprocal connections with the cerebral cortex via the cortico-pontocerebellar and cerebello-thalamocortical pathways, supporting bidirectional information flow. Significantly, these circuits are not restricted to motor processing: posterior, cognition-related cerebellar regions also rely on the same anatomical routes. Feedforward projections arise from motor and prefrontal areas, including the dorsolateral prefrontal cortex, and target cerebellar lobules IV to VI, crus II, and lobule X. In turn, feedback projections from the dentate and interposed nuclei return to the motor and prefrontal cortices. 16 17 18 19 Functional connectivity studies 20 21 further validate these pathways, underscoring the cerebellum's critical role in higher-order cognition in addition to motor coordination.

There are direct and indirect interconnections between the cerebellum and the basal ganglia ( Figure 1 ). Retrograde tracing studies in primates 22 have revealed disynaptic projections from the subthalamic nucleus to the cerebellar cortex. At the same time, other investigations 23 24 25 have demonstrated dentate efferents projecting to the striatum, globus pallidus, and substantia nigra. These interconnected loops contribute not only to motor control, but also to reward-based learning and language functions. 26 27 28

The cerebellum also connects with other subcortical regions involved in cognition and emotion. Direct projections to the ventral tegmental area modulate reward and social behavior; optogenetic activation of this pathway enhances social interactions in mice. 29 Connections between the hippocampus and the cerebellum have been identified in animal models and in humans, linking cerebellar activity to spatial memory and navigation. 30 31 Interactions with the hypothalamus involve histaminergic and orexinergic pathways, integrating stress responses and emotional regulation with cerebellar control. 32 33 34 Furthermore, direct projections from the dentate nucleus to the amygdala have been demonstrated, with clinical studies on PD 34 35 showing associations between this circuit's activity and anxiety severity. These cerebello-amygdalar pathways may also contribute to social cognition. 36

Recently, Hadjiosif et al. 37 reported that the cerebellum is crucial for long-term procedural memory, although it is not required for short-term memory. Just as the medial temporal lobe serves as a gateway for declarative memories, the cerebellum acts as the gateway for persistent sensorimotor memories, reinforcing the concept of distinct neural substrates for memories of different durations. 37

In a seminal longitudinal study with 20 patients with isolated cerebellar lesions, Schmahmann and Sherman 4 described the constellation of symptoms now recognized as CCAS. The deficits reported included executive dysfunction—encompassing planning, organization, abstract reasoning, verbal fluency, and working memory—along with perseverative thinking, inattention, visuospatial disorganization, impaired visuospatial memory, and language difficulties such as dysprosody, agrammatism, and mild anomia. Personality changes were also common, ranging from affective blunting to disinhibited and inappropriate behavior, frequently accompanied by emotional lability. These disturbances were attributed 3 4 to the disruption in the cortico-pontocerebellar pathways linking associative and paralimbic cerebral regions with the cerebellum. The authors 3 4 proposed that, just as the cerebellum regulates the force, rhythm, and accuracy of motor activity, it also modulates the speed, consistency, and appropriateness of mental processes, thereby contributing to nonverbal communication and the regulation of mood and unconscious motivation.

Cerebellar cognitive affective syndrome aligns with Barkley's 38 EF theory. Barkley's behavioral inhibition model 38 39 further explores the cerebellum's role in behavior regulation. According to this model, EF is crucial to regulate emotions, engage in social behavior, and control impulses, whether in speech or movement. It is involved in initiating and inhibiting behaviors, verbal reasoning, problem-solving, action planning, sequencing, self-awareness, cognitive flexibility, decision-making, and motor control. Barkley 39 noted that behavioral inhibition provides the time necessary for executive processes to occur and for intended actions to be evaluated before execution. When inhibitory pathways are impaired, errors and misjudgments become more likely, as seen in frequent behaviors in patients with CCAS.

In addition to behavioral inhibition and motor control, Barkley's EF model 39 includes four core executive domains crucial for cognitive functioning: working memory (integrating time awareness, self-reflection, and prospective/retrospective thinking); affective self-regulation (driven by intrinsic motivation and stimulus salience); internalized speech (enabling reflective processing and behavioral modulation); and reconstitution (analyzing verbal and nonverbal behavior). Behavioral inhibition underpins executive processes and contributes to motor control, fluency, and syntax. 38 39 40 41 42

Executive function should not be viewed as a single, localized brain function; instead, it involves a network of interrelated brain regions that enable adaptive behavior by supporting flexible responses to environmental demands. Executive functioning requires high-level cognitive processing that influences affective, motivational, behavioral, and social domains. 42 Neuropsychological and functional neuroimaging studies have demonstrated that the cerebellum plays an active role in modulating EF through its connections with prefrontal and paralimbic areas. This cerebellocortical interaction is fundamental to optimize cognitive function and enhance the fluency and efficiency of cognitive and emotional processing. 43 Corticocerebellar dysfunction—particularly involving posterior cerebellar connections with parietal and temporal cortices—may disrupt the integration of sensory, spatial, and mnemonic information. 44

Koziol, Budding, and Chidekel 8 examined the interaction involving movement, thought, and EF, focusing on the cerebellum's role. They proposed that the brain evolved primarily to control action rather than for cognition alone, and they called for a redefinition of EF within a framework of continuous sensorimotor interaction with the environment. The authors 8 argued that behavioral control is central to EF and explored the cerebellum's critical role in this process. Procedural learning supports the acquisition of declarative knowledge, and these systems interact through sensorimotor anticipation—establishing a direct link involving movement, thought, and embodied cognition. 8

The understanding of EF and its relationship with the cerebellum, along with contributions from Barkley 45 and Uehara et al., 42 underscores the complexity and multifunctionality of the human brain. Executive function emerges as a key component of behavioral and cognitive adaptation, transcending the traditional view of its location in the prefrontal cortex. Historically linked to motor control, the cerebellum is now recognized as an active contributor to EF regulation, highlighting the integrated nature of brain function. This expanded perspective suggests that therapeutic and educational interventions targeting EF disorders may benefit from incorporating the cerebellum's role and connectivity with other brain regions. 42 43 45

## INSIGHTS FROM CLINICAL FINDINGS

Several neurodegenerative disorders manifest cognitive impairment in association with cerebellar dysfunction ( Table 1 ). In SCAs, distinct cognitive profiles have been documented across subtypes, with variability in the severity and nature of deficits. These differences, although sometimes subtle, carry important clinical and prognostic implications, underscoring the need for systematic neuropsychological evaluation in affected patients. 11 The role of genetic factors in cognitive dysfunction has also become increasingly evident through investigations examining the relationship between cytosine-adenine-guanine (CAG) repeat length and cognitive impairment. 11 46 47 While some studies demonstrate correlations regarding repeat length and specific cognitive domains, others suggest the relationship is complex and may be modulated by additional genetic and environmental factors. This variability underscores the importance of a multifaceted approach when evaluating cognitive risk in patients with SCAs. A review on repeat expansion diseases 10 highlights that SCAs are associated with widespread cognitive deficits, except for language. While vocabulary, comprehension, and repetition (basic language functions) are generally preserved, language components dependent on EFs—such as category-based verbal fluency, complex syntactic construction, and set-shifting—may be impaired.

**Table 1:** Neurodegenerative diseases with evidence of cerebellar involvement in cognition

Disease	Main cognitive findings
SCA1 50 51	Selective deficits in attention, nonverbal intelligence, and memory; rapid cognitive decline in longitudinal studies.
SCA2 50 51 72 74	Broad impairment: executive functions, immediate and delayed verbal memory, attention, and social cognition.
SCA3 49 50 53 57	More pronounced deficits in delayed memory and executive functions; abnormal connectivity in prefrontal and temporoparietal networks.
SCA6 51 58	Traditionally considered a “pure ataxia”, but studies show executive slowing, visuospatial deficits, and reduced verbal fluency.
SCA7 51 52	Selective deficits, especially in nonverbal tasks; impairment in social cognition.
SCA10 60 61	Executive and visuospatial deficits, sometimes associated with psychiatric features (e.g., psychosis).
SCA17 59	Among the most severe subtypes, it often evolves into a dementia-like syndrome due to diffuse corticosubcortical degeneration.
SCA19/22 62 63	Cognitive profile often mirrors CCAS: executive dysfunction, visuospatial memory deficits, and impulse control issues.
SCA21 64	Typically, early onset, combined cognitive and behavioral changes (executive, memory, and social regulation deficits).
SCA36 65	Preserved global cognition, but selective executive dysfunction and prominent apathy.
DRPLA 66	Dementia and psychiatric manifestations, including psychosis.
Huntington's disease 12 67 70	Deficits in working memory, visuospatial skills, emotion, and social cognition are linked to cerebellar involvement.
Parkinson's disease 83 84 85	Abnormal cerebello–prefrontal connectivity; deficits in working memory, attention, and socioemotional cognition.
Alzheimer's disease 87 88 89	Progressive cerebellar involvement in advanced stages; atrophy in crus I/II; and visuospatial and navigation deficits.

Abbreviations: CCAS, cerebellar cognitive affective syndrome; DRPLA, dentatorubral-pallidoluysian atrophy; SCA, spinocerebellar ataxia.

The most frequently affected cognitive domains in SCAs include EFs, attention, and processing speed. Multiple studies 48 converge on the presence of a mild-to-moderate dysexecutive syndrome in patients with cerebellar ataxias, characterized by cognitive slowing, impaired mental flexibility, and deficits in memory retrieval. In a case series 48 including patients with the SCA2, SCA3, SCA6, and SCA14 subtypes, all individuals demonstrated slowed processing speed, reduced mental flexibility, and impaired free recall. At the same time, specific abilities such as object naming (semantic language) and the consolidation of new memories remained preserved. 10 48 49 In addition to fronto-executive dysfunction, many patients with SCAs experience deficits in sustained and divided attention, as well as working memory. 50 Episodic memory alterations vary by subtype: encoding and free recall may be impaired, while recognition memory is often preserved, suggesting a deficit in retrieval rather than storage. 49

Verbal memory deficits may also be prominent in SCAs. Ma et al. 50 studied 18 patients (8 with SCA1, 2 with SCA2, and 8 with SCA3) and matched healthy controls. They found that SCA2 patients exhibited significant impairments in immediate and delayed memory, while SCA3 patients demonstrated greater deficits in delayed memory, with relative preservation of immediate recall. Interestingly, SCA1 patients did not show significant impairments in verbal memory, suggesting subtype-specific memory profiles. These findings support that memory deficits in SCAs follow distinct patterns depending on the subtype, with SCA2 presenting broader impairment of the amnesic system and SCA3 showing selective deficits in consolidation or retrieval after a time interval. 49 50

Moriarty et al. 51 investigated the cognitive and clinical progression of patients with different SCA subtypes; 13 patients with CAG repeat expansion SCAs (types 1, 2, 3, 6, and 7) were evaluated through a comprehensive battery of cognitive and mood assessments at 2 time points, with a mean interval of 7.35 years. The clinical scores worsened significantly over time, indicating disease progression. The most common neuropsychological impairments included deficits in EF, information processing speed, attention, visual memory, and theory of mind-related personality features. Distinct cognitive decline trajectories were observed across different SCA subtypes, with SCA1 showing the most rapid cognitive deterioration, and SCA6, the slowest. Minimal mood changes were reported, and no correlation was found between cognitive decline and magnetic resonance imaging (MRI)-based cerebral atrophy. The authors concluded that cognitive decline is a distinct and subtype-dependent feature of the SCA phenotype in addition to worsening motor symptoms. Furthermore, cognitive decline may occur regardless of the severity of the cerebellar or extracerebellar motor symptoms. 51

Sokolovsky et al. 52 examined the cognitive and social profiles of 8 patients with SCA1, SCA2, and SCA7. Using an extensive neuropsychological and social cognition battery, they identified significant subtype-specific differences. Patients with SCA2 exhibited the most pronounced deficits, particularly in attention, nonverbal intelligence, and EF; The SCA1 patients showed relatively-preserved cognitive function, with some nonverbal intelligence and attention impairments; and the SCA7 patients demonstrated selective impairments, primarily affecting the nonverbal subtests of the Wechsler Adult Intelligence Scale–Revised (WAIS-R).

Regarding social cognition, difficulties in emotion attribution were observed in patients with SCA2 and SCA7 but not in those with SCA1. However, one SCA1 patient exhibited deficits in theory of mind tasks. These findings suggest that cognitive and social impairments may result from disruptions in cerebellocortical and limbic system connections. Each SCA subtype may present with distinct cognitive and social profiles depending on the extent and location of the cerebellar damage. 52

Other research into social cognition in SCAs 51 53 has also revealed impairments in theory of mind and emotion recognition tasks. These deficits likely stem from disruptions in cerebellocortical circuits that support social information processing, expanding the cognitive phenotype beyond memory and EFs. A longitudinal study 51 showed that deficits in social perception may emerge as the disease progresses. This aligns with the concept of CCAS, which posits that affect and social behavior are modulated by the cerebellum. 48

In a comprehensive review, Lin et al. 54 highlighted the prevalence of impulsivity, emotional lability, and behavioral changes across multiple SCA subtypes. These neuropsychiatric symptoms—often falling within the CCAS spectrum—reflect dysfunctions in corticolimbic and frontocerebellar circuits, with a significant impact on overall cognitive functioning. 54 D'Agata et al. 55 assessed sustained attention and adaptive behavior in SCA patients, documenting marked difficulties in maintaining attention over time and associated functional impairments; a qualitative analysis revealed behavioral dysregulation in social settings, likely secondary to cerebellar-related cognitive disorganization. More recently, studies 11 56 have examined how cognitive dysfunction affects daily functioning and quality of life. Cognitive impairments—particularly in EF and processing speed—are significant predictors of functional independence and quality of life in patients with SCAs, often more so than motor impairments. 56

Braga-Neto et al. 57 evaluated 38 Brazilian patients with SCA3 using an extensive cognitive and behavioral assessment to explore the presence of CCAS; although their findings revealed significant impairments in visuospatial function and EF, as well as affective symptoms consistent with elements of CCAS, the authors could not definitively conclude that the full syndrome was present. This limitation stems from the fact that SCA3 involves widespread extracerebellar neurodegeneration, including basal ganglia and cortical regions, making it difficult to isolate the specific contribution of cerebellar pathology. Nevertheless, the observed pattern of deficits aligns closely with those attributed to disruption of cerebrocerebellar circuits.

Although disruptions occur across multiple neural networks, cognitive impairment is a consistent feature of diseases with cerebellar degeneration. Neuropsychological studies 58 have also identified that SCA6 patients may present with slowing of visuospatial and executive processing, as well as inferior performance in phonemic verbal fluency tests and verb generation. This finding is interesting, since SCA6 is traditionally considered a “pure” ataxia, limited to the cerebellum. Subtype 17 is among the most severe, often leading to a dementia-like syndrome due to widespread corticosubcortical degeneration. 59 Subtype 10 has been linked to deficits in executive and visuospatial domains, occasionally accompanied by psychiatric features such as psychosis. 60 61 In SCA19, cognitive dysfunction frequently mirrors the profile of CCAS, with impairments in EF, visuospatial memory, and impulse control. 62 63 Subtype 21 typically begins early in life and is characterized by combined cognitive and behavioral alterations, including deficits in EF, memory, and social regulation. 64 Patients with SCA36 often maintain preserved global cognition, though selective executive dysfunction and prominent apathy are reported. 65 Finally, patients with dentatorubral-pallidoluysian atrophy (DRPLA) may present dementia and psychiatric manifestations, including psychosis. 66

In addition to cerebellar ataxias, other neurological disorders are also characterized by cerebellar degeneration and cognitive impairment, a relationship that has been documented in the literature. In HD, the cerebellum and its pathways contribute to the modulation of these non-motor features. 12 Scharmüller et al. 67 demonstrated that anger recognition deficits in HD correlate with cerebellar atrophy, while other studies 68 have described broader impairments in facial emotion recognition. Although some reports link these alterations to hippocampal dysfunction, 69 converging data suggest that cerebellar involvement also plays a role in socioemotional deficits. Overall, the cognitive profile of HD arises from widespread corticosubcortical pathology. However, the cerebellum appears to exert modulatory influences on working memory, visuospatial abilities, and emotional processing. Thus, progressive cognitive decline in HD may ultimately reflect the combined disruption of cortical and cerebellar circuits. 12 67 68 69 70

## NEUROIMAGING INSIGHTS

The structural characterization of cerebellar degeneration and its relationship to cognitive manifestations have been enhanced through the use of advanced neuroimaging techniques. Guo et al. 71 investigated structural degeneration patterns in SCAs using diffusion-tensor imaging (DTI) and volumetric analysis, identifying significant correlations between cerebellar white matter integrity and performance on visual memory and planning tasks. Although the study 71 primarily focused on motor aspects, the observed structure–function correlations support the hypothesis that specific cerebellar substrates directly contribute to cognitive deficits in patients with hereditary ataxias, providing neuroanatomical evidence for the pathophysiological basis of these alterations.

Subtype 2 is the most consistently associated with cognitive impairment. Cerebellar degeneration and its associated pathways result in deficits in EF, memory, language, and visuospatial processing, all of which negatively impact quality of life. 8 11 In a study involving 8 SCA2 patients and matched healthy controls, Bürk 72 employed functional MRI (fMRI) and neuropsychological testing, including intelligence quotient (IQ), attention, EF, and verbal and visuospatial memory, and found that executive dysfunction was a prominent and early feature of the disease. Verbal memory deficits were also identified, although to a lesser degree than executive impairment.

Complementary neuroanatomical evidence has emerged from advanced neuroimaging studies that have revolutionized the understanding of the structural basis of cognitive dysfunction in SCAs. Combining morphometric analyses and diffusion-tensor connectivity in 15 patients with SCA2 and 15 healthy controls, Hernandez-Castillo et al. 73 identified significant structural and microstructural alterations in the posterior cerebellum. The volume reduction observed in lobules crus I, II, and VI, along with compromised white matter integrity in the superior and middle cerebellar peduncles, correlated directly with deficits in planning, working memory, and attentional shifting. These data prove that dysfunction in corticocerebellar circuits contributes significantly to the cognitive impairments observed in SCA2. 73

Clausi et al. 74 also aimed to characterize social cognition deficits in 13 patients with SCA2 and explore their neurobiological basis by comparing them with matched healthy controls. The patients underwent several social cognition tests, including the Reading the Mind in the Eyes Test, the Emotion Attribution Test, and the Faux Pas Recognition Test. A brain MRI protocol assessed cerebellar gray matter (CGM) atrophy via voxel-based morphometry and microstructural changes in cerebellar peduncles using DTI. The SCA2 patients showed impairments in immediate perceptual components of mental state recognition (such as identifying thoughts and feelings from eye expressions), emotion attribution (such as anger), and understanding of false beliefs. A reduction in CGM volume was observed in lobules IX, VIIIb, and the crus II—regions implicated in social cognition processing—behavioral performance correlated with CGM reduction and microstructural abnormalities in the cerebellar peduncles. 74

Ye et al. 53 employed resting-state fMRI to investigate functional alterations in neural networks related to mental state processing in individuals with SCA3. The results demonstrated abnormal functional connectivity in temporoparietal and prefrontal regions, structures critically involved in inferring the thoughts and intentions of others. These alterations suggest that the social impairment observed in SCA3 may be related to specific dysfunctions in higher-order cerebellocortical networks, thereby expanding our understanding of the neurobiological mechanisms underlying social cognition deficits in this population. 53

Dopaminergic pathways have long been implicated in behavioral regulation, particularly in reward-based learning, but increasing evidence also supports their contribution to higher-order cognitive processes. Dopamine is thought to modulate hierarchical interactions within frontostriatal circuits, influencing executive control and adaptive behavior. 75 However, this relationship is limited in certain cerebellar disorders. In a study using [99mTc]-TRODAT-1 SPECT, Braga-Neto et al. 75 investigated patients with SCA3 and found no correlation between striatal dopamine transporter (DAT) density and a range of cognitive deficits. These findings suggest that cognitive dysfunction in SCA3 is unlikely to result primarily from frontostriatal dopaminergic disruption. 75

In HD, neuroimaging and clinical studies have consistently demonstrated structural and functional alterations in cerebellar regions that correlate with cognitive and behavioral outcomes. 12 Azevedo et al. 70 found that higher gray matter density in the posterosuperior lobe was associated with mood symptoms in HD, while motor worsening and better cognitive performance also correlated with posterior cerebellar changes. Importantly, higher Montreal Cognitive Assessment (MoCA) scores were linked to increased density in left lobule VIII, a region implicated in sensorimotor processing and working memory.

Voxel-based morphometry studies further supported cerebellar involvement: Galvez et al. 76 demonstrated correlations between early cognitive-behavioral deficits and extrastriatal structures, including the cerebellum. Functional MRI investigations reinforced this view: Wolf et al. 77 reported cerebellar hypoactivation during working memory tasks, whereas Georgiou-Karistianis et al. 78 observed robust cerebellar activation in premanifest and early HD, which was absent in symptomatic patients. Additional evidence extends to emotion processing and learning-related functions, with studies 79 80 showing abnormal cerebellar engagement during affective and visuospatial tasks.

Cerebellar perfusion abnormalities have also been described. Deckel et al. 81 noted increased blood flow in the cerebellum but decreased flow in the caudate during cognitive activation, suggesting vascular dysregulation may contribute to oxidative stress in HD. Comparative analyses between HD and primary cerebellar degeneration provide further insight: Brandt et al. 82 reported that while both conditions feature executive dysfunction, HD is distinguished by pronounced spatial and amnestic deficits, underscoring the contribution of cortical involvement in addition to cerebellar changes.

In PD, emerging neuroimaging evidence has reinforced the notion that the cerebellum plays a critical role in the cognitive disturbances. Functional imaging studies have consistently demonstrated abnormal cerebellar connectivity with prefrontal networks. In patients with mild cognitive impairment, resting-state fMRI revealed disrupted connectivity between the dorsolateral prefrontal cortex (DLPFC) and the cerebellum, with weaker connectivity correlating with poorer performance in working memory tasks. 83 Similarly, emotional processing tasks have shown that patients with impaired recognition of emotional vocal cues present with more severe lesions to the right cerebellar hemisphere, 84 highlighting its role in socioemotional cognition.

Positron-emission tomography (PET) imaging has further delineated distinct patterns of cerebellar metabolism in PD. Hypermetabolism in the anterior lobe and vermis correlates with motor dysfunction severity, whereas increased activity in the right crus I and crus II associates with cognitive deficits. 85 These findings suggest that different cerebellar territories contribute selectively to motor versus cognitive domains. Moreover, meta-analytic data confirm that patients with social cognition deficits exhibit increased activation in the posterior cerebellum, 86 possibly reflecting compensatory mechanisms that attempt to offset frontostriatal dysfunction.

Additionally, in AD, neuroimaging studies have increasingly shown that the cerebellum, although relatively spared in the early stages, becomes progressively involved as the disease progresses. Voxel-based morphometry analyses 87 have revealed focal atrophy in the bilateral crus I and II regions, which are closely connected to cortical areas that also undergo severe degeneration, reinforcing the notion of disrupted cerebellocerebral circuits. Fluorodeoxyglucose-PET studies 88 in large AD cohorts further support this, showing crossed cerebellar diaschisis with reduced metabolism in the right cerebellum and contralateral temporal-occipital cortices, highlighting maladaptive connectivity as the disease progresses. Evidence 89 also suggests degeneration of the inferior olivary nucleus, which reduces excitatory input to the cerebellum and may contribute to the spatial navigation difficulties that often emerge early in dementia.

## TREATMENT INSIGHTS

Although early research has demonstrated the feasibility of cognitive rehabilitation in cerebellar disorders, developing SCA-specific protocols has been gradual. Currently, tailored cognitive rehabilitation strategies for SCAs remain limited, with no standardized approaches developed exclusively for these disorders. Most available interventions still focus primarily on motor symptoms and physical function, highlighting a critical gap in the management of cognitive aspects. Nonetheless, several promising approaches have been explored to address cognitive deficits and improve the quality of life in these patients. 54

Ciancarelli et al. 90 conducted one of the earliest systematic investigations on cognitive rehabilitation, assessing 24 individuals with Friedreich's ataxia for 1 year. The participants underwent three distinct hospital-based rehabilitation periods, which included cognitive-behavioral therapy. The results showed stability in most neuropsychological measures and specific improvements in the Stroop test and short-term memory, suggesting that cognitive interventions may exert a protective effect against cognitive decline. The authors 90 concluded that, when combined with neuromotor rehabilitation, cognitive training could be an effective strategy for the comprehensive care of patients with hereditary ataxias.

Giehl et al. 91 92 93 published a series of studies evaluating the effects of a home-based working memory training program delivered through the digital platform NeuroNation (Synaptikon GmbH) in cognitively-preserved patients with PD. In the first randomized clinical trial, 91 the intervention led to increased neural efficiency, evidenced by reduced brain activation and reorganization of functional connectivity—particularly during tasks requiring working memory manipulation. These neural changes correlated with improvements in neuropsychological test performance, indicating a beneficial cognitive impact even in the early stages of the disease; in a subsequent study 92 using the sensitive “What was where” visuospatial memory task, they observed significant improvements in accuracy after 5 weeks of training, with effects sustained at the 14-week follow-up. Together, these studies suggest the feasibility and efficacy of NeuroNation as an early and personalized cognitive intervention for PD. While studies on digital cognitive training in SCA patients remain scarce and inconclusive, 62 Vieira et al. (personal communication) have submitted a study showing that patients with SCA2 demonstrated notable improvements in memory using the same platform tested by Giehl et al. 91 92 93 in PD.

Non-invasive brain stimulation (NIBS) has emerged as a promising therapeutic approach to modulate cerebellar and prefrontal networks. Techniques such as transcranial magnetic stimulation (TMS), repetitive TMS (rTMS), and transcranial direct current stimulation (tDCS) have been investigated 94 for their potential to restore functional connectivity and improve cognition. A comprehensive review by Pope and Miall 95 highlighted that disruption of pathways such as the dentatorubral circuit contributes to cognitive deficits in disorders like Friedreich's ataxia. They argued that enhancing cerebello-prefrontal connectivity may help rebalance these circuits, with possible benefits in EF, language, working memory, and affective regulation.

Clinical applications have begun to support this rationale. In patients with SCA3, Wu et al. 96 conducted a double-blinded, placebo-controlled trial showing that 15 sessions of low-frequency cerebellar rTMS over 3 weeks led to significant cognitive gains, including an average 2.4-point increase on the MoCA, modest improvements on the Mini-Mental State Examination (MMSE), and reductions in anxiety and depression. Similarly, Farzan et al. 97 reported that a patient with late-onset idiopathic cerebellar atrophy showed improvement in mobility, dual-tasking, and cognitive performance after 21 sessions of cerebellar TMS, accompanied by neurophysiological evidence of enhanced excitability in both motor and prefrontal regions. These findings suggest that rTMS can promote plasticity within cerebellocortical networks, translating into cognitive as well as motor benefits, particularly in CCAS.

Importantly, cerebellar stimulation has also shown therapeutic potential in AD. In one study, 98 rTMS applied to crus II improved multiple cognitive domains, including memory, executive function, and visuospatial skills, underscoring the pathological involvement of cerebellar circuits in AD and the promise of neuromodulation as a strategy to mitigate cognitive decline.

## Final considerations

The evidence supporting the cerebellum's role in cognition has become increasingly compelling. Beyond animal and neuroimaging studies that map cerebellar structures and their connections, clinical observations provide powerful confirmation that degenerative changes in the cerebellum and its pathways are associated with cognitive dysfunction—even in conditions such as AD. Future investigations will help clarify the extent to which memory and other cognitive functions are linked to cerebellar mechanisms, moving beyond the traditional view that cerebellar dysfunction is limited to executive processes. Likely, forthcoming research will further elucidate the cerebellum's contribution to social cognition and emotion, advancing our understanding of this still enigmatic structure.
